# Teleradiology and the Compensation Conundrum in India

**DOI:** 10.7759/cureus.51342

**Published:** 2023-12-30

**Authors:** Harpreet Grewal, Niharika Prasad, Gagandeep Dhillon, Rahul Kashyap

**Affiliations:** 1 Radiology, Florida State University College of Medicine, Pensacola, USA; 2 Radiology, Tata Motors Hospital, Jamshedpur, IND; 3 Internal Medicine, University of Maryland Medical Center, Glen Burnie, USA; 4 Medicine, Drexel University College of Medicine, Philadelphia, USA; 5 Research, Harvard Medical School, Boston, USA; 6 Research, Global Remote Research Program, Saint Paul, USA; 7 Critical Care Medicine, Mayo Clinic, Rochester, USA; 8 Research, WellSpan Health, York, USA

**Keywords:** radiologist workload, digital imaging, healthcare outcomes, compensation, teleradiology

## Abstract

Teleradiology, an established telemedicine practice globally, has significantly enhanced the accessibility of high-quality radiological interpretations in remote areas worldwide, especially in India, thereby improving healthcare outcomes. The utilization of teleradiology services has seen a marked increase, expanding its reach into more distant regions of the country. However, this burgeoning field faces numerous regulatory, financial, and technical challenges. The current scenario regarding teleradiology in India is a double-edged sword. There is an increasing demand for it due to the expanding population, resulting in larger imaging volumes needing radiologist services. This editorial aims to examine the prevailing challenges in teleradiology in India, with an emphasis on the compensation model for teleradiologists, which has not kept pace with the growing demand for services and often remains inequitable.

## Editorial

The advent of teleradiology in India has been instrumental in extending radiology services to the nation's most remote corners [1]. The demand for radiology reporting services has consistently risen throughout the country, driven by increased imaging volumes and the need for specialized interpretations. Technological advancements in the past decade have significantly widened its scope, enabling the digital transmission of images from remote locations to teleradiologists anywhere in the country [2]. This capability has greatly enhanced the quality of care in these regions, especially highlighted during and after the COVID-19 pandemic [3].

However, the business model underpinning these teleradiology services has sparked considerable debate, especially regarding the interpretation of scans from other countries by radiologists trained in India. This practice has catalyzed a surge in teleradiology services, outsourcing work from abroad to radiologists in India at nominal reimbursement rates [4]. While this may be a lucrative business model for teleradiology companies at the onset, it may not be entirely beneficial for radiologists whose compensation remains modest compared to their international counterparts [5]. This may lead to teleradiologists opting to interpret an unusually large number of scans in each shift. It may result in errors of omission and/or commission, thus adversely affecting quality and patient outcomes.

While the expansion of teleradiology services undoubtedly enhances healthcare outcomes in remote areas, it is crucial to maintain the quality of radiological interpretations and continue attracting top talent to the field [3]. The trend of employing radiologists in India for reading international scans at lower reimbursement rates does not support this objective and can lead to job dissatisfaction, increased workloads, and burnout [6]. The benefits of these technical advancements should ideally translate into improved, accessible, and affordable healthcare for patients, as well as higher job satisfaction for the radiologist.

The teleradiology setup offers significant advantages for radiologists, allowing them to work for multiple employers across India from the comfort of their homes [7]. However, despite the critical role that accurate and timely radiological reports play in patient care, the current reimbursement rates for teleradiologists in India are disappointingly low. These rates do not reflect the effort involved in correctly interpreting radiology scans, nor do they consider the extensive training and certification exams these professionals undergo [8]. Fresh residency graduates start high-volume teleradiology jobs for supplementary income in addition to a full-time job. All these factors may lead to diminishing the perceived value of radiology as a profession among other clinicians. Many government hospitals and private centers are equipped with CT scanners, but there is a scarcity of qualified and proficient radiologists. This may lead to the reporting of suboptimal images or images lacking essential clinical data, motivated primarily by financial gains.

A stark disparity exists in the payment models between Indian and foreign-trained radiologists, particularly in comparison to Western-trained radiologists. An Indian radiologist specialized in cross-sectional imaging, five years postresidency training, would usually be earning approximately 60,000 USD per year; in contrast, a similarly qualified radiologist in the USA would be earning approximately 350,000 USD per year [8]. Teleradiologists in India are often engaged to provide preliminary reads for Western countries, but the compensation they receive is markedly lower than what their Western peers earn for similar services. The ethical implications of this practice have been questioned by entities like the American College of Radiology (ACR), yet it persists [9]. This practice may not only undermine the global reputation of Indian-trained radiologists but may also demand that they adapt their working hours to foreign time zones. When comparing the practice of teleradiology in India with that of the US, a significant advantage emerges for teleradiology companies employing radiologists in India, primarily attributable to the difference in time zones. A radiologist in India can report scans during their daytime hours, which correspond to nighttime in the US, potentially offering more effective reporting than a US radiologist working during nocturnal hours [8]. While India has a lower cost of living compared to the US, it should not justify lower reimbursements. The demanding nature of radiological study interpretations, requiring intense work and judgment, is consistent regardless of location, warranting equitable compensation.

The issue extends beyond international scans. Within India, the reimbursement model for radiologists is also suboptimal. National teleradiology providers sometimes offer as little as Indian National Rupee (INR) 350-450 ($5-6) for interpreting a contrast CT or MRI exam, a complex task with significant implications for patient outcomes [10]. Such practices can lead to lower quality interpretations, radiologist burnout, and consequently, poorer patient outcomes. It is even more important as the value of the care and patient outcomes are at the core of the newly launched Ayushman Bharat universal health coverage in India, expanding imaging services to an even wider patient population [11].

The current trend of cost-cutting at the expense of the radiologists is unsustainable and risks creating a dissatisfied teleradiology community. This could hinder innovation in teleradiology and slow its expansion. It is imperative to advance the specialty and provide sustainable career opportunities for new graduates. With the current dismal reimbursement rates, there is an imminent risk of failing to attract the best talent into radiology, which would significantly impede the growth of the specialty.

The emergence of artificial intelligence (AI), cloud-based Picture Archiving and Communication System (PACS) systems, and other advancements underscore the fact that teleradiology is not just the future, but a necessity [12]. However, concurrently, the intrinsic value of teleradiology as a specialty may have been diminished, with a greater emphasis on its viability as a business model rather than recognizing its critical role in maintaining high-quality reports to enhance patient care. This represents a significant disconnect, as teleradiologists are often geographically distant from the patients they serve and rarely have direct interaction with the referring healthcare providers. Therefore, the responsibility falls upon the radiologists to demonstrate their clinical expertise and contribute meaningfully to patient care.

In conclusion, it is crucial for the radiology community to unite in establishing standardized reimbursement rates for teleradiologists that accurately reflect the value and effort involved in producing high-quality radiology reports. Acknowledging the issue of inadequate reimbursement as a significant concern, organizations such as the Indian Radiological & Imaging Association (IRIA) have put forth recommendations regarding fair market value compensations for teleradiologists [13]. However, it appears that these recommendations are largely disregarded. The radiology community must stand together to reject undervalued compensation in the interest of job satisfaction and patient outcomes.
